# Second order motion compensated spin echo cardiac diffusion tensor imaging on clinical MR systems

**DOI:** 10.1186/1532-429X-18-S1-P61

**Published:** 2016-01-27

**Authors:** Christian T Stoeck, Constantin von Deuster, Sebastian Kozerke

**Affiliations:** 1 grid.5801.c0000000121562780Institute for Biomedical Engineering, University and ETH Zurich, Zurich, Switzerland; 2 grid.13097.3c0000000123226764Imaging Sciences and Biomedical Engineering, King's College London, London, UK

## Background

Recently second order motion compensated spin echo (SE) sequences in conjunction with high performance gradient systems have been proposed for diffusion weighted (DWI) [1] and diffusion tensor imaging (DTI) [2, 3] of the human in vivo heart. The method allows for free breathing acquisition without requiring dedicated patient feedback systems or other provisions [4], facilitating the transition of cardiac DTI into a clinical environment.

In this preliminary study we investigate the limits of SE based cardiac DTI relative to available gradient strengths to explore the required gradient specifications of clinical MR equipment.

## Methods

Cardiac DTI was acquired on three healthy volunteers (28 ± 4 years/67 ± 11 kg) on a 1.5T clinical system (Philips Healthcare, Best, The Netherlands) equipped with a 5-channel cardiac receiver coil and a high performance gradient system (G_max_ 80 mT/m, slew rate 100 mT/m/ms). The imaging sequence consists of a second order motion compensated SE imaging module with a single shot EPI readout [2]. The sequence parameters are: FOV 230 × 100 mm^2^, resolution 2.5 × 2.5 mm^2^, slice thickness 6 mm, 10 averages, TR 3R-R. Three slices (apex/mid/base) were acquired triggered to 50% systole during free breathing and navigator gating (gating window 7 mm). G_max_ per channel was set to 30, 40, 60 and 80 mT/m resulting in a TE of 96, 85, 73 and 66 ms. Three orthogonal diffusion encoding directions with b = 100 s/mm^2^ and 9 directions with b = 450 s/mm^2^ were acquired. The orientation of the directions was optimized to generate effective gradient strengths of 43, 56, 84 and 105 mT/m.

The LV was manually segmented and helix as well as transverse angles [5] were estimated. For a sector wise comparison the LV was segmented according to the AHA sectors with 5 transmural layers. To estimate the influence of low SNR due to prolonged TE, angulation was estimated additionally using only 4 out of 10 averages acquired at G_max_ = 80 mT/m.

## Results

**Figure 1:**
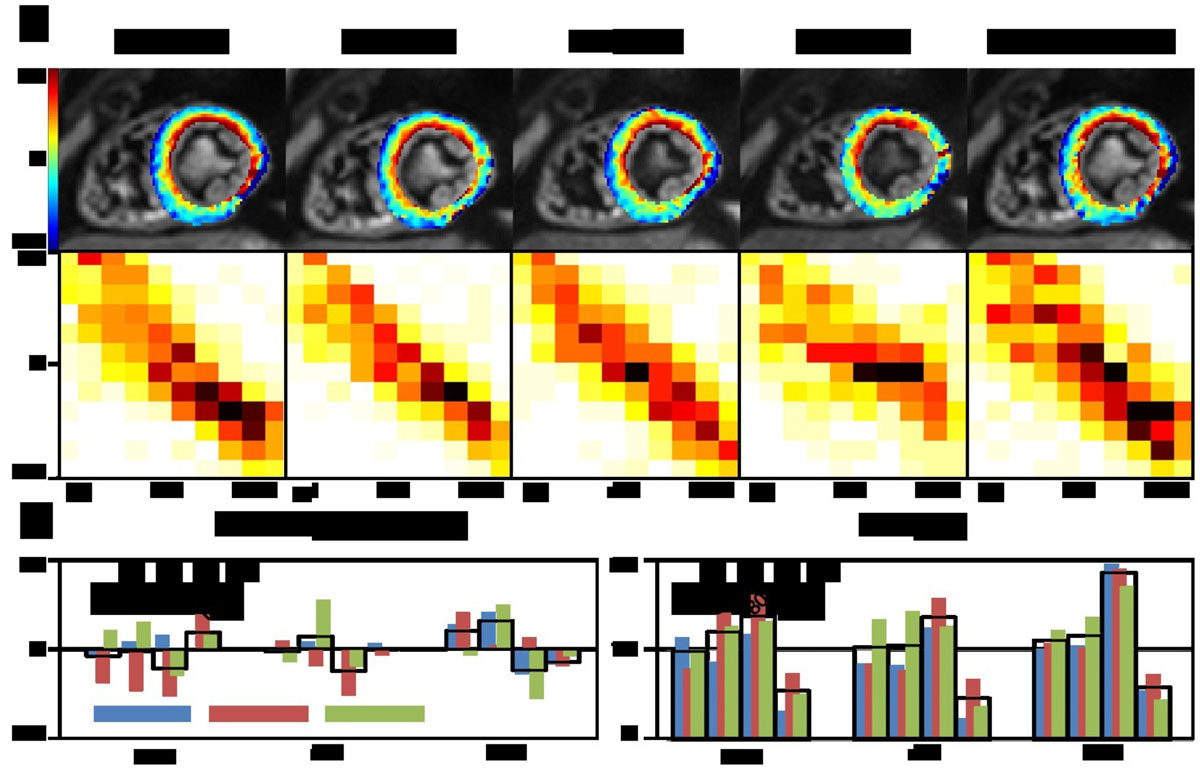
**a) Helix angle maps and transmural helix angle histograms of a midventricular slice**. G_max_ per channel is varied from 30 mT/m to 80 mT/m. Data reconstructed from only 4 signal averages at Gmax = 80 mT/m mimicking reduced SNR of the low G_max_ acquisition is shown in the right column. b) Signed mean differences and RMSE for a sector wise analysis at apical, midventricular and basal level. The black boxes indicate the average over all sectors.

**Figure 2:**
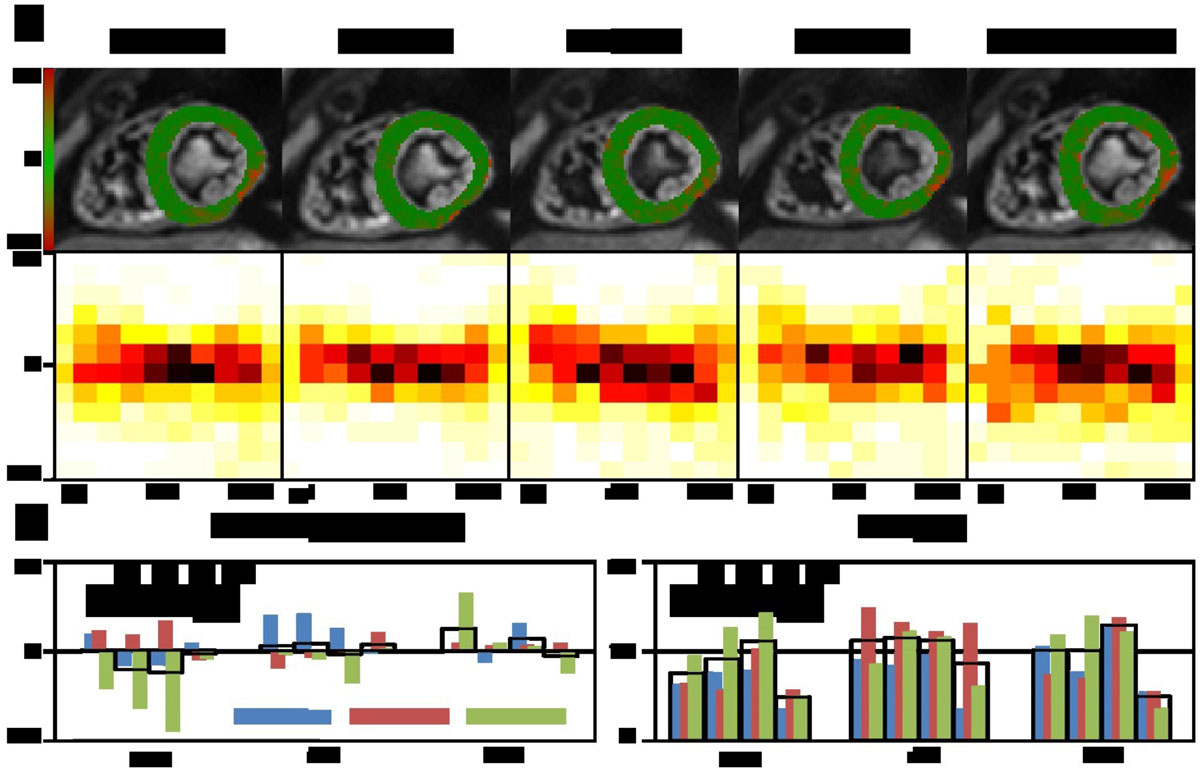
**a) Transverse angle maps and transmural transverse angle histograms of a midventricular slide**. Gmax per channel is varied from 30 mT/m to 80 mT/m. Data reconstructed from only 4 signal averages of Gmax = 80 mT/m mimicking reduced SNR of the low Gmax acquisition is shown in right column. b) Signed mean differences and RMSE for sector wise analysis at apical, midventricular and basal level. The black boxes indicate the average over all sectors.

Figure 1a) shows example helix angle maps at mid-ventricular level and the corresponding transmural helix angle histograms. Image quality was found to be comparable for G_max_ as low as 40 mT/m. Figure 1b) shows the sector wise comparison based on singed mean difference and root-mean-squared errors (RMSE). Figure 2 presents the corresponding analysis for the transverse angle. Large transverse angle are predominantly found at the border of the myocardium and in the vicinity of the posterior vein. The average RMSE was below 12° over all slices for helix and transverse angles for G_max_ as low as 40 mT/m. No trend is visible from the signed mean differences indicting a potential bias. The increased patchiness of the angle maps found with lower G_max_ is attributed to reduced SNR.

## Conclusions

This study indicates that second order motion compensated spin echo diffusion tensor imaging is feasible on clinical MR systems without dedicated high performance gradients.
